# Supplementary figures and images for: Developing an observation protocol for online STEM courses
Source: PLoS One. 2024 Jan 25;19(1):e0297359. doi: 10.1371/journal.pone.0297359 (PMC10810430; doi:10.1371/journal.pone.0297359)

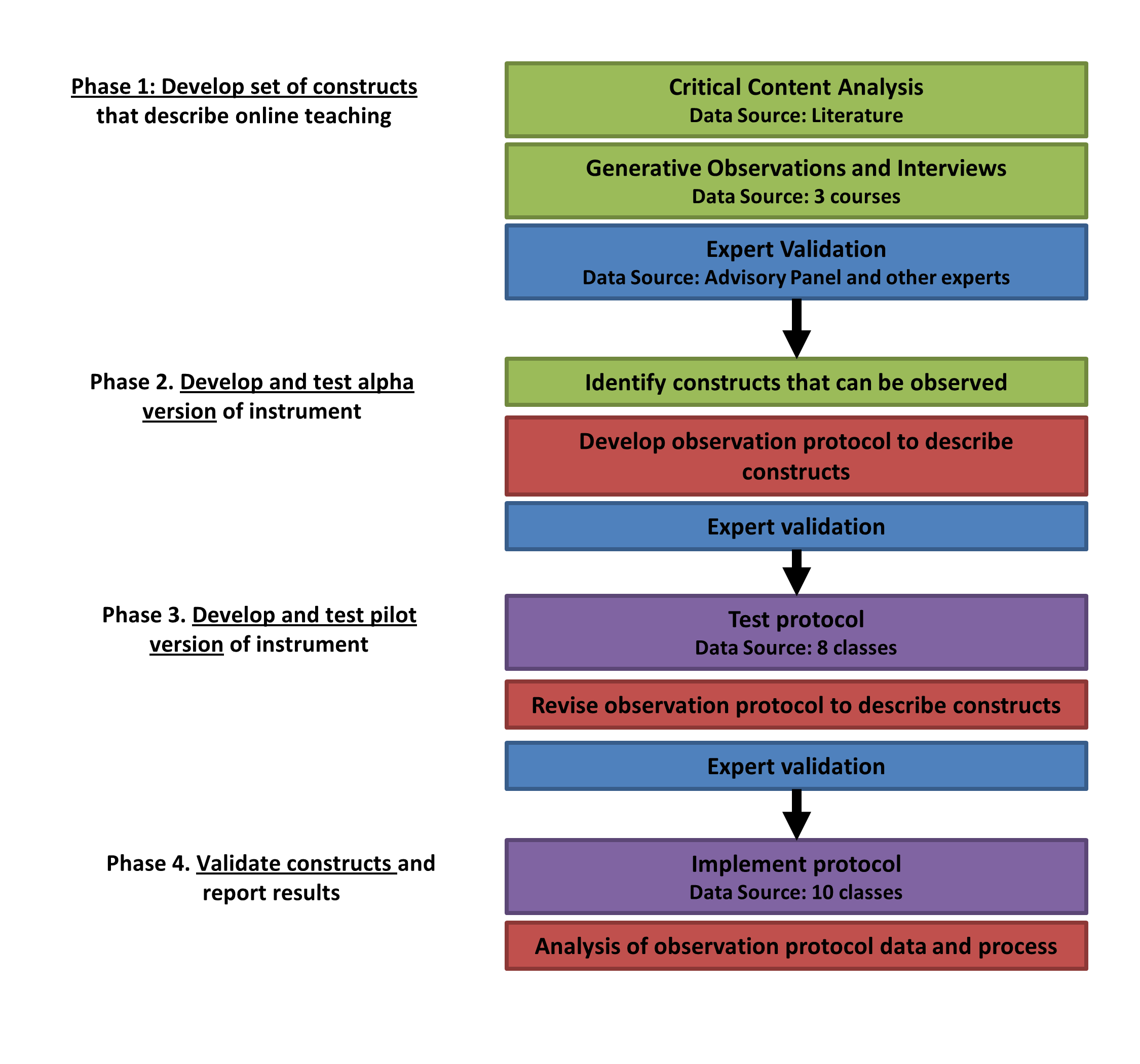

Supplement: S1 Appendix — (TIF) [file pone.0297359.s002.tif]

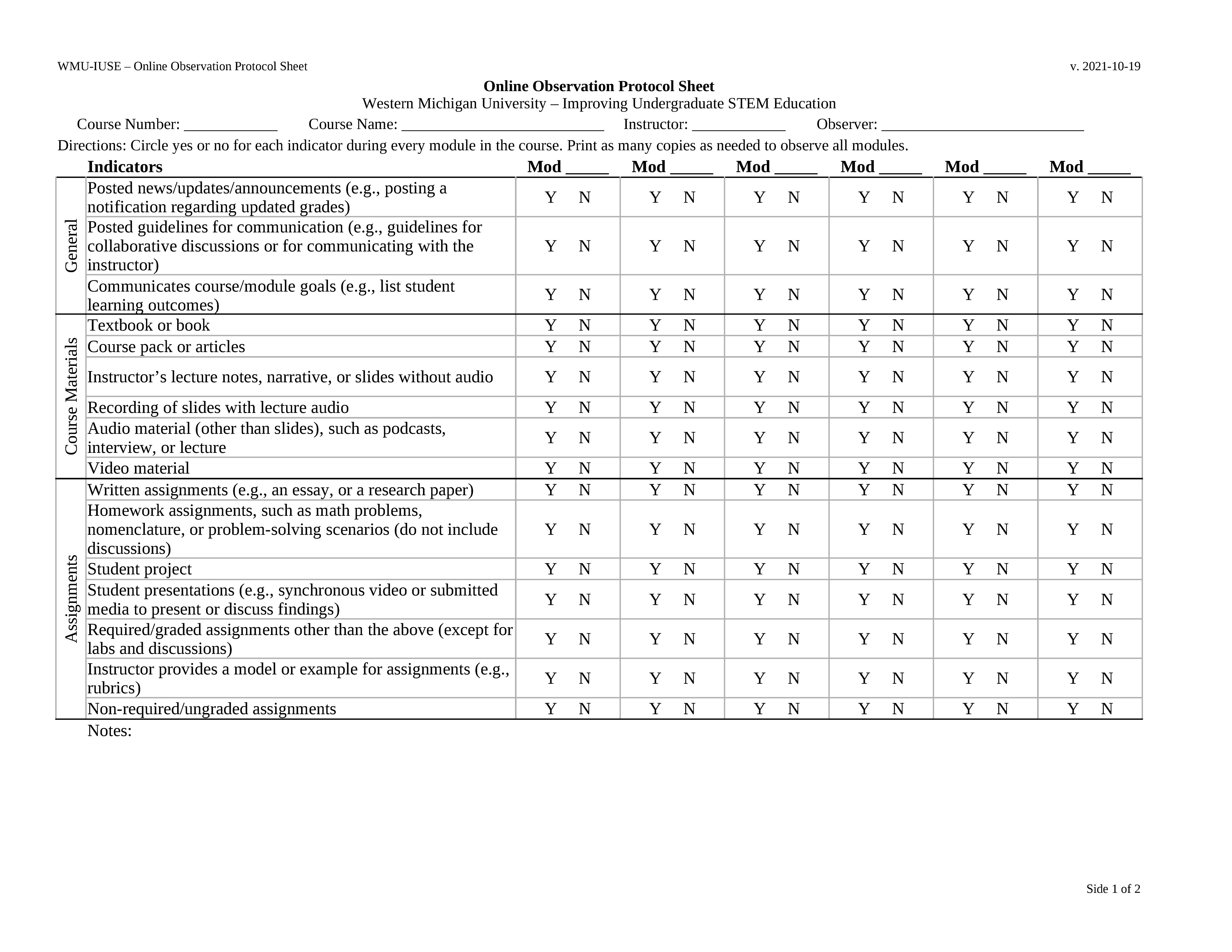

Supplement: S2 Appendix — (ZIP) [file pone.0297359.s003.zip › IUSE Observation Protocol_Page_1.tif]

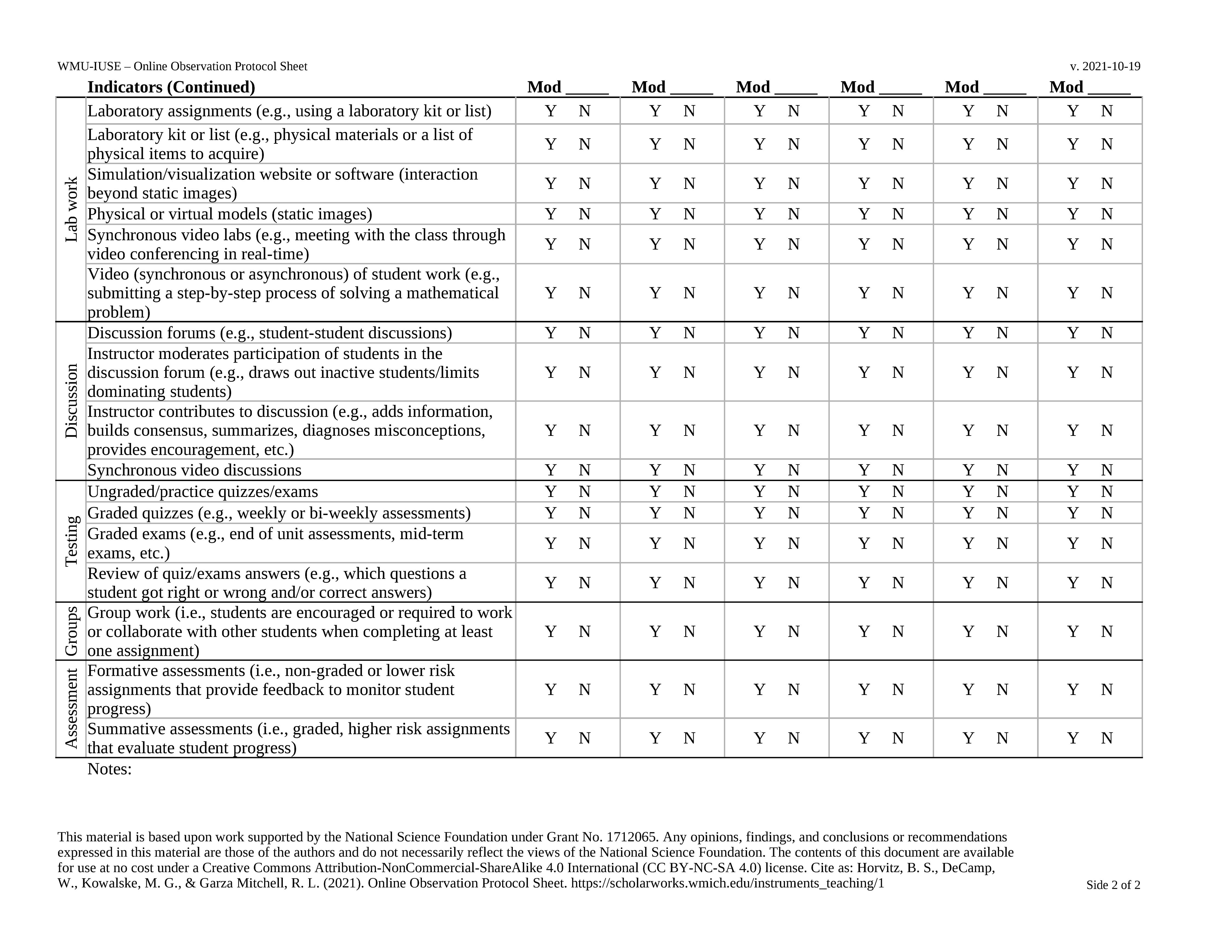

Supplement: S2 Appendix — (ZIP) [file pone.0297359.s003.zip › IUSE Observation Protocol_Page_2.tif]
