# Supplementary material for: Developing an observation protocol for online STEM courses
Source: PLoS One. 2024 Jan 25;19(1):e0297359. doi: 10.1371/journal.pone.0297359 (PMC10810430; doi:10.1371/journal.pone.0297359)
Supplement: Attachment — Submitted filename: Response to Reviewers.docx [file pone.0297359.s004.docx]

PLOS Editors,

Thank you for your time and efforts in reviewing our manuscript. Please find below our responses to each point raised by the reviewers and an explanation for how we addressed each point. The reviewers’ points are copied below in italics.

Sincerely yours,

Brian Horvitz, corresponding author

*1. Abstract: the implications of the study should be stated in the abstract. “The research team recommends reconsidering the inclusion of some items with low levels of agreement or continued revisions on such items.” What does this sentence refer to? Please elaborate it.*

We have revised the abstract to more clearly state the implication as such; that this instrument should be further developed and used for a variety of purposes. We also further considered the statement about reconsidering the inclusion of some items and deleted it because we did not feel it reflected the contents of the manuscript accurately.

*2. Introduction: The introduction cannot adequately justify this study.*

*I suggest that it could be improved in several ways. First, it could cite more recent sources to support the claims about the growth of online STEM courses, as well as the lack of accurate methods to measure online instruction. Second, the authors may define “accurate” in this study. “Accurate” is a very subjective and ambiguous word. it could clarify what constitutes an observational protocol and how it differs from other instruments or tools for describing and measuring instructional practices. Third, it could specify the intended audience and scope of the project, such as whether it focuses on a particular discipline, level, or type of online STEM course.*

We have reviewed the manuscript and added additional citations to recent works where appropriate. We have also removed the word “accurate” in a place where its meaning was, as noted, subjective. There are other uses of the word in the manuscript that are aspirational rather than judgmental, and we did not remove those. We have also specified that the instrument was developed to be used across all STEM disciplines.

*3. Literature review:*

*3.1 The logic in the “Existing Measurement Instruments for STEM Classrooms” was loose. For example, the authors listed some instruments (face-to-face instruments) in the first and second paragraph. Then, in the third paragraph, the authors stated, “Although the adoption of online learning approaches is increasing across higher education (Johnson, Adams, Becker, Estrada, & Martín, 2013), there is still a need for instruments that measure online teaching practices.” In my humble opinion, I cannot see a strong connection between paragraph 2 and paragraph 3. The authors did not introduce the current online instruments for STEM courses.*

*I suggest a comprehensive review of instruments for teaching practices in both traditional classrooms and online courses are needed.*

Providing a comprehensive review of instruments would require substantial text and lengthen the manuscript considerably. Additionally, providing a full review of existing instruments is a project onto itself, and would be beyond the scope of the present study. However, there are existing works that have provided reviews of other instruments, and we have added a citation accordingly.

*3.2 In addition, the literature should be updated. Some selected studies are not the latest.*

We added citations to Cameron et al., 2021; Morgan, 2020; Baldwin and Trespalacios, 2017; and Castellanos-Reyes, 2020.

*3.3 there are some confusing terms need further explanation. For example, the second paragraph on page 4, “Significant effort by instructional designers, faculty developers, and online platform providers have provided checklists and rubrics of best practices (e.g., Quality Matters Higher Education Rubric, BlackBoard Exemplary Course Program Rubric, MERLOT Evaluation Standards for Learning Materials, Online Learning Consortium*

*Quality Scorecard). The terms in parentheses need to be explained, because they are difficult to understand for readers who see these terms for the first time.*

For each of the instruments listed, we have added citations so readers can learn more about their origin. We also wrote out what MERLOT stands for.

*3.4 The framework of CoI. Section of “Conceptual Framework for Describing Online Instruction” should be improved.*

*3.4.1. On Page 4, the authors should provide a clear definition of what they mean by effective online teaching practices and how they operationalize them in their instruments. They should also explain how the CoI framework aligns with their definition and operationalization of effective online teaching practices.*

We agree the use of the word “effective” was a poor choice and it was removed.

We changed “We used the Community of Inquiry (CoI) framework to guide our understanding of teaching throughout the process developing our instruments.” to “We used the Community of Inquiry (CoI) framework to guide our understanding of teaching throughout the process developing our instruments.”

*3.4.2. A more recent and relevant literature that supports the validity and reliability of the CoI framework for measuring online teaching practices are strongly needed. The authors should justify why they chose to use the CoI framework over other frameworks or models that have been proposed for online teaching practices. They should also discuss how their instruments address the gaps or challenges that have been identified in previous studies using the CoI framework.*

In the manuscript, we are applying a long standing and widely used educational literature that has not been heavily used for online learning. We consider our work to be on the forefront of this. Also, we suggest that theoretical frameworks are not measured for validity and reliability, so we disagree that more CoI literature that related to the framework’s validity and reliability is unnecessary.

*3.4.3. The authors should avoid making general claims or implications based on the CoI framework without providing empirical evidence or data from their own study or other studies. They should also acknowledge the limitations and potential biases of their instruments and the CoI framework. The reason of “the framework has been extensively used, including in two special issues of the Internet and Higher Education in 2010” are not convincing.*

We have carefully reviewed the manuscript with these comments in mind, and made revisions in multiple places to ensure that any claims are supported by either the present study or cited works. We have also added a discussion about bias and other limitations to the discussion section.

*3.4.4. The last paragraph (p6) in the literature review section should be put in “Methods”. The reasons of choosing mixed research methods should be further elaborated.*

The paragraph was moved as suggested. We also added more details about the methodological approach used, with a citation pointing to more details.

*4.1 I suggest adding a figure showing the iterative process.*

We have added a figure as suggested.

*4.2. “four courses were observed”, what are the four courses? Detailed information is needed.*

We have revised the description (in the phase 1 section) to note the field/area of the courses.

*4.3. Do authors think the subjects in STEM courses will influence the observation items?*

The limitations section we added (in response to a comment above) acknowledges that further refinements would be helpful and require further testing in additional subjects.

*5. Results*

*The results section presents the agreement rates among observers for each item in the observation protocol. It is expected that each indicator could be explained in the section 4. What does these indicators refer to in the STEM courses.*

Because there are 38 items listed, it would be a very lengthy addition to provide definitions for each. Additionally, any definitions added at this point would likely be misleading, as they were not provided during observations and thus would not necessarily be reflective of the meaning ascribed to the items during data collection. We have added some text to the manuscript that notes the goal to have items on an instrument such as this one to be as self-explanatory as possible.

*6. Discussion*

*The discussion section provides a thoughtful analysis of the implications of the results for the future design and control of online STEM courses. However, it would be helpful to provide more information on how the protocol aligns the framework of CoI and how the current study relates to previous studies. Literature should be cited in the discussion section.*

We have added the following to the manuscript: “As described above, the teaching presence element of the CoI framework has three components (Garrison & Arbaugh, 2007): instructional design and organization, facilitating discourse, and direct instruction. Through the four phases of this instrument development process, particularly during the critical content analysis process, instrument items were created that reflect each of these three components. The use of expert panel review in phases 1, 2 and 3 along with the overall observer agreement rate of 77.4% (with variation between sets of observers, as described above) provide evidence that the final version of the the observational protocol will give its users, be they practitioners or researchers, online teaching data that reflects key components of the CoI framework.”

*However, the article seldom mentioned the reason why the items were grouped in 7 criteria, namely General, Course Materials, Assignments, Activities, Lab work, Discussion and Testing. More literature can be added to support the use of the 7 criteria.*

These terms were assigned for ease of use by the end-users. These terms emerged iteratively through the instrument design process. We have noted in the manuscript that this is for ease of use by the observers.

*Besides, the four phases were not clearly defined in the article. In the abstract, they were put as “research, design, pilot-testing, refinement and implementation of the protocol.” In Line 163 to 216, the authors use different terms to explain them. It is advised to align with the team and clearly define the 4 phases. The authors may use a figure to illustrate this iterative process.*

The phrase quoted from the abstract does not refer to the four distinct phases. We are willing to revise this language if deemed necessary, but we currently believe the language is clear.

We have added a figure to illustrate the process as requested.
