# Supplementary material for: Developing an observation protocol for online STEM courses
Source: PLoS One. 2024 Jan 25;19(1):e0297359. doi: 10.1371/journal.pone.0297359 (PMC10810430; doi:10.1371/journal.pone.0297359)
Supplement: Attachment — Submitted filename: Response to Reviewers 120723.doc [file pone.0297359.s005.doc]

Developing an observation protocol for online STEM courses

Reviewers' Comments & Author Responses

PLOS Editors,

We offer our thanks to the reviewers for their suggestions. Our specific responses to each of the comments are detailed below. Changes are all tracked in the submitted, revised manuscript.

Sincerely yours,

Brian Horvitz, corresponding author

Reviewer #3: 1. Row 34, please notice the use of brackets; Row 100, please notice the format. Row 203, add “,” after “completed”. Row 212, “each of the ten courses was”.

These corrections have been made.

2. Please notice the concept of teaching presence when talking about the CoI framework. The description here, in rows 122 to 123, “instructors and students interact with each other and with course content,” is more connected to teaching presence and social presence. If social presence is also an important concept for the research?

Garrison, Anderson, & Archer (1999) define social presence as “the ability of participants in a community of inquiry to project themselves socially and emotionally, as ‘real’ people (i.e., their full personality), through the medium of communication being used.” Likewise, Garrison & Arbaugh (2007) define social presence as “the ability of learners to project themselves socially and emotionally, thereby being perceived as ‘real people’ in mediated communication.” Based on these definitions, we would consider social presence to be beyond the scope of the present study and unable to be addressed with the data collected here.

3. The elements of teaching presence from 169–170 may be deleted to make this part clearer.

This revision has been made to the manuscript.

4. Can the number of participants or observations of the courses in phrase 4 be informed?

We have revised the phase 4 section to more clearly explain the number of observations.

5. In the result part, can statistic analysis, for example, the chi-square test or t test, or other methods be used to examine the reliability or consistency of the results of two observers?

The interrater reliability test used (the percentage of observations recorded the same between observers) is the standard test used. The data used are binary, so a t-test (which assumes continuous variables) would not be appropriate. A chi-square would not be appropriate because the data only allow for a 2x1 table (a 2x2 table could be constructed using the raw answers rather than agreement rates, but that would be far less precise because the observations would not be matched in such a configuration). We have revised the text to indicate in the result section that the interrater reliability test used here is the customary and appropriate test.

6. The section “Introduction” should be more powerful to emphasize the importance and significance of the development of the observation protocol.

We have revised the introduction to more strongly state the gaps in the literature and resources that this study and protocol fill.

7. There should be more connection with the former research in the section "Discussion". It may be from the perspective of, for example, the validation of the design or processing.

Language was added at the end of the “Discussion” section that refers back to literature cited in the “Instrument development and methods” section that speaks to the validation of the instrument design.

8. Content from rows 275 to 287 may be in the section “limitations and future”.

This revision has been made to the manuscript. We omitted the word “future” given Reviewer #4’s recommendation to include a conclusion section.

9. After the construction of the observation protocol, it should be displayed in the main body, and each section should be explained. Although the protocol is mainly developed from the CoI framework, the origin of each section should be referenced and explained well.

The observation protocol is designed as a worksheet, so including it in the main body would take up considerable space and disrupt the flow of the text. Additionally, each observation item is included in Table 1, so the relevant text is already provided to readers adjacent to the relevant text. We have added some text regarding the development of sections to Phase 2.

10. The definition of “STEM course” is different from science subject courses, such as math, geoscience, and biology. Thus, please clarify the definition of “STEM course” in the section “Introduction” or “Literature Review”. At the same time, please check if the references related to STEM courses or STEM education are correctly referenced.

Because there is no universally agreed upon list of disciplines encompassed in “STEM,” we developed our own list of disciplines for purposes of recruiting instructors and courses for this study: biology, chemistry, environmental sciences, geosciences, and mathematics & statistics. Some disciplines that would fall under the STEM umbrella were not included because courses were not yet being offered online at the university used for this study. We are hesitant to add this definition to the introduction or literature review, as relevant literature uses differing sets of disciplines and we would not want to suggest to readers that our literature review is restricted just to the disciplines we use for our own data collection (or would we find it desirable to interrupt the flow to discuss each study’s definition/disciplines to clarify). Therefore, we have added this clarification to Phase 1, where we discuss the selection of courses for our study.

Reviewer #4: The article has made general adjustments in response to the reviewers' suggestions. However, a few minor issues still need attention. First, it is recommended that the writing of the Review of Literature section be revised to Literature Review.

This revision has been made to the manuscript.

Also, the Conclusion section was not found in the article, so it is suggested that a Conclusion section be added, or that the two be combined to change the subtitle to Conclusion and Discussion.

We have revised the subsection accordingly.

Furthermore, there are some editorial concerns within the article, so it is kindly requested that the article format be carefully reviewed and refined.

We have carefully reviewed the manuscript to identify and correct any errors.
